# Investigating whether shared video-based consultations with patients, oncologists, and GPs can benefit patient-centred cancer care: a qualitative study

**DOI:** 10.3399/bjgpopen20X101023

**Published:** 2020-04-01

**Authors:** Theis Bitz Trabjerg, Lars Henrik Jensen, Jens Sondergaard, Natacha Dencker Trabjerg, Jeffrey James Sisler, Dorte Gilså Hansen

**Affiliations:** 1 Research Fellow, Research Unit of General Practice, Department of Public Health, University of Southern Denmark, Odense, Denmark; 2 Chief Physician, Department of Oncology, Lillebaelt University Hospital, Vejle, Denmark; 3 Associate Professor, Danish Colorectal Cancer Center South, Center of Clinical Excellence, Vejle Hospital, Institute of Regional Health Research, University of Southern Denmark, Vejle, Denmark; 4 Professor, Research Unit of General Practice, Department of Public Health, University of Southern Denmark, Odense, Denmark; 5 Research Fellow, Department of Oncology, Lillebaelt University Hospital, Vejle, Denmark; 6 Professor, Department of Family Medicine, Faculty of Health Sciences, University of Manitoba, Winnipeg, Canada; 7 Associate Professor, Research Unit of General Practice, Department of Public Health, University of Southern Denmark, Odense, Denmark

**Keywords:** neoplasms, general practice, video consultation, patient-centred care, framework method, oncologists

## Abstract

**Background:**

Guidelines have proposed that GPs should have a central role as coordinators of care and support patients with cancer during all stages of treatment, follow-up, and rehabilitation. Multidisciplinary video consultation involving the patient with cancer, the oncologist, and the GP may help to define roles and tasks, and this resulting clarity may enable greater support for patients with cancer.

**Aim:**

To explore the consultation structure, content, and task clarification when a GP and an oncologist are attending a video consultation with a patient with cancer.

**Design & setting:**

A qualitative study took place in the Region of Southern Denmark to investigate multidisciplinary video consultations, based on thematic analysis.

**Method:**

Recordings of 12 video consultations were analysed using the framework method. A combined deductive and inductive approach was undertaken. The deductive themes were selected based on a consultation guide given to the doctors before the consultations.

**Results:**

The study identified 15 themes, which were grouped into the following three categories: the implications of sharing a consultation; consultation structure; and health concerns.

**Conclusion:**

Multidisciplinary video-based consultations with a patient and two health professionals succeeded in having a patient-centred communication style. In clarifying tasks between the GP and oncologist to support the patient, work-related issues and professional support for psychosocial challenges were always a task for the GP. Dissemination of this first-line evidence may improve acceptability among medical specialists and help assist GPs in supporting patients with cancer. However, focus on the involvement of relatives should be emphasised.

## How this fits in

A new type of multidisciplinary consultation may improve continuity of cancer care for patients. Video can bring together the patient, the GP, and the oncologist, leading to multidisciplinary communication that can be patient-centred. Knowledge exchange, task clarification, and further GP involvement are rendered possible by this type of consultation.

## Introduction

Bringing the patient with cancer together with the oncologist and GP by means of video consultation may be an effective method of supporting the patient.

The World Health Organization suggests that cross-sectoral learning and collaborative practice will contribute to the delivery of safe, effective, and coordinated cancer care.^1^ Guidelines have proposed that GPs should have a central role as coordinators of the course of treatment and be more involved in supporting patients with cancer.^2–5^ However, cancer follow-up and rehabilitation still pose considerable challenges for GPs during and following long-term, hospital-based cancer treatment.^2,6,7^ Unmet needs regarding physical and psychosocial problems are frequent.^8–10^ Patients may experience difficulties related to uncertainy caused by whether their GP or oncologist is responsible for providing and coordinating care.^11^

In the Netherlands, joint consultations, where the GP physically accompanies the orthopaedic patient for a consultation with a specialist at the hospital, have demonstrated a reduced number of follow-up appointments, fewer tests and investigations, and improved health status.^12^ A study in which GPs participated in cancer multidisciplinary team (MDT) meetings in Belgium showed that GPs could contribute to better continuity. However, such consultations are not possible in everyday clinical practice for practical and economic reasons, and the authors suggest the use of video conferences.^13^ Video consultation can overcome the logistical barriers of bringing together professionals who are not in the same room.^14,15^ Furthermore, the literature shows that patients can experience effective doctor–patient communication through video-based consultations and can establish a good relationship with specialists.^14,16–18^

However, little is known about completion of video consultations with the patient, oncologist, and GP. The authors argue that the usual consultation structure and way of communication may be challenged, preventing a patient-centred approach. Patient-centredness has been defined by Epstein *et al* as engaging the patient, responding to patients’ emotions, assessing the desire for information, and checking for understanding, including framing information regarding decisions.^19^ Therefore, this qualitative study of video-based, tripartite multidisciplinary consultations aims to explore the consultation structure, health concerns, and patient-centredness when two doctors are attending consultations through video with a patient with cancer at the offices of oncologists or GPs.

## Method

This study is designed and reported according to the Standard for Reporting Qualitative Research (SRQR) guideline.^20^

### Context

The qualitative work is part of a randomised controlled trial (RCT) entitled 'The Partnership Study', which evaluates shared video consultations between the patient with cancer, the oncologist, and the GP. It aims to improve patient-perceived intersectoral cooperation, continuity of care, patient-centredness, and healthcare needs.^21,22^ The RCT aims to include 278 adults receiving oncological treatment. The design of the RCT has been described in more detail elsewhere.^23^

**Figure 1.**
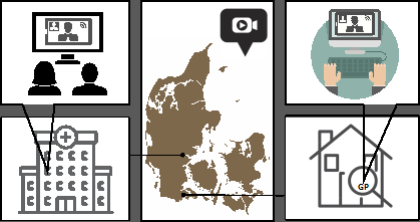
Schematic illustration of a video consultation with the patient at the hospital together with the oncologist. The GP is taking part in the consultation by means of video from their clinic located close to the patient’s residence.

The Partnership Study was carried out at the Department of Oncology, Vejle Hospital, Denmark and included the participating patients’ GPs.^23^ In Denmark, GPs are located in their own local offices close to where patients live (Figure 1). All citizens have access to free medical service in primary care and at public hospitals.^24^ The consultations were conducted as part of the planned standard programme at the hospital, but in case a patient chose to be located at the GP’s office, an additional consultation was scheduled.

As part of the intervention, doctors were handed a short consultation guide, including the overall aim of the intervention and a list of potential themes for the consultation (Box 1). The consultation guide was inspired by the Calgary-Cambridge Guide, supporting doctor–patient communication training.^25^ Furthermore, the guide was discussed and received feedback from a user panel of GPs and oncologists.

Box 1 **The list of potential themes included in the consultation guide presented for the GPs and oncologists before the consultations.**

- A summary of the patient trajectory
- Patient concerns and desire for the consultation
- Sharing knowledge regarding comorbidity
- Psychosocial resources and needs
- Agreements on who should take care of what and when in the future
- Physical wellbeing
- Medicine
- Psychological wellbeing
- Relatives
- Ability to work
- Late complications and side effects to the treatment
- Other

### Units of study

The study features recordings of consultations of 12 people with cancer aged 36–71 years (seven female and five male), which took place between October 2018 and February 2019. Two patients were treated for colorectal cancer, six for lung cancer, one for gynaecological cancer, one for breast cancer, and two for pancreatic cancer at the Department of Oncology, Lillebaelt Hospital, Denmark. Four received oncological treatment with a potentially curable aim (one breast, one gynaecological, one lung, and one colon cancer) and eight had accepted palliative treatment (five lung, one colon, and two pancreatic cancer). Eight different oncologists (three male and five female) and eleven GPs (five male and six female) participated in the consultations. The oncologists were all medical oncologists (four chief specialists and four staff specialists), and all GPs were specialist in family medicine with an average length of service of 13 years (4-30 years). In three cases, the patient was located at the GP’s office; the remaining patients were at the hospital, where a nurse and relatives also attended. The average consultation time was 18.5 minutes (8–32 minutes).

### Sampling strategy

The sampling was started when the recording of the consultations became technically achievable in October 2018. The goal was to include cases representing different clinical and organisational characteristics. The study followed the principle for saturation during the analysis described by Vasileiou *et al;* ^26^ for example, when no new themes emerge, and themes start to repeat often, a saturation point may be reached. Therefore, at this point it was decided to stop the inclusion of data. By random, it ended with a consecutive sample.

### Data collection methods

**Figure 2.**
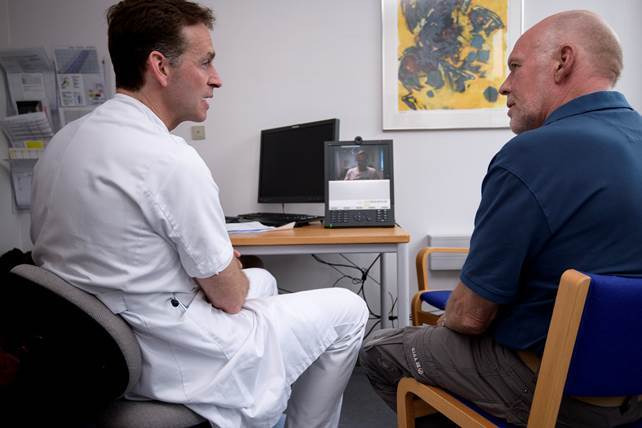
Picture of the video consultation with the patient at the hospital, together with the oncologist. The GP is taking part in the consultation by means of video from his clinic located in the rural region (picture-in-picture feature is turned off in this picture). Reprinted with permission from the Danish Medical Journal, photographer Palle Peter Skov.

The consultations were conducted by use of video through a virtual meeting room with a picture-in-picture feature (Figure 2). For research purposes, digital recordings were stored. The recordings showed the speaking participants in a full-screen picture and the other participants in thumbnails. Furthermore, demographic data on the participants were collected by surveys and electronic patient records and stored in a secure RedCap^27^ database established for the RCT.

### Data collection instruments and technologies

The GP and oncologist joined the virtual meeting room using a unique code. The recording started at first entrance.

At the department level, the Cisco, TANDBERG E20 screens already in use at the hospital were used. The GPs used the Cisco Jabber system with an external combined microphone and speaker and a small webcam attached on top of their computer screen.

For technical support, one of the authors was present at the GPs’ offices before the consultations, but did not participate in the consultations.

### Data processing

The Region of Southern Denmark hosted the virtual meeting room and stored the video recordings initially on its secure server. The shared consultation was recorded from the start to the end.

The video recordings were audio-transcribed verbatim by the company Translateplus. The transcripts were used for citations only and did not include behavioural content such as gestures and use of technology. Individual names were anonymised. For data analysis, video recordings were used; the verbal audio information was the focus for the analysis, however, which therefore did not include content such as gestures, facial expressions, and non-verbal communication.

### Qualitative approach and research paradigm

The analytical work was based on the recordings; that is, by looking at them on the screen thoroughly and listening to what was said. The framework method described by Gale *et al* was used as a systematic way to draw descriptive and explanatory conclusions clustered around themes.^28^ To begin the analytical process, a first version framework matrix was created. The columns represented cases and the rows included all themes of the consultation guideline (Box 1) and others covering the structure of the consultation.^28^ A combined deductive and inductive approach was used, and new rows were added as themes appeared during the analytical process (Box 2).

Box 2 **Description of the stages in the analytic process. Stages 1–5 were performed by the first and last authors, and stage 6 by all authors involved.**

1. The first and last author familiarised themselves with the data by viewing some recordings, separately making notes in parallel. Thereby becoming familiar with the data.
2. The first and last author developed the first framework matrix by first reviewing three video-recordings together, taking notes separately in parallel. By comparing these notes, the first framework matrix was created.
3. Separately, the first and last author applied the first framework matrix by viewing five recordings.
4. It was decided to revise the matrix by adding new themes based on a consensus meeting after applying the first matrix.
5. After having analysed all the recordings sitting apart with the revised matrix, the first and last author met to discuss the findings. In the absence of consensus, the recordings were reviewed together and discussed. Thereby, the final framework matrix was generated and used for interpreting the data.
6. The first author summarised the themes that had emerged into three categories. Details regarding categories were discussed with all co-authors during the writing process.

### Researcher characteristics and reflexivity

TBT and DGH were the principal researchers responsible for the analysis. TBT is a PhD student and GP trainee. DGH is a doctor and associate professor with a long career in general practice research. JS and JJS are general practice professors also working as general practitioners. LHJ is an associate professor and chief specialist in oncology. NDT is a PhD student in training for oncology.

### Data analysis

The principal researchers were responsible for making the framework matrix and performed the analysis. The transcripts were referred to for accuracy. Six stages were followed for the analysis, and then the interpretation^28^ (Box 2).

### Techniques to enhance trustworthiness

Member-checking was used to enhance the trustworthiness of the framework matrix. One of the authors was the oncologist for three of the cases and agreed on the content of the matrix applied to the consultations.

## Results

Based on the analysis of 12 consultations, 15 themes were identified and grouped into three categories:

1. the implications of sharing a consultation;
2. consultation structure; and
3. health concerns.

### Category 1: the implications of sharing a consultation

Four themes defined this category: ‘professional learning’, ‘articulation of the GP’s knowledge and expertise’, ‘shared decision making’, and ‘clarification of tasks and responsibilities'.

#### ​Professional learning

Information passed from the oncologist to the GP and vice versa, most often from the oncologist to the GP. The information functioned as learning points for the doctors and was related to the specific patient, but in several cases addressed in general terms. Technical medical terms were only used for interprofessional learning between doctors for short discussions, and not in connection with patient communication. Topics included vaccination when treated with chemotherapy or immunotherapy, medical treatment of pain and side effects of oncological treatment such as nausea, treatment of comorbidity, and follow-up programmes at the Department of Oncology:

*​'How about, I was thinking about a pneumococcal vaccine for pneumonia.'* (GP4)

*​'I believe it* [pain in feet] *doesn’t have much to do with cancer treatment.'* (GP10) *'No, I totally agree with you. I do not think it has.'* (Onco10)

#### ​Articulation of the knowledge and expertise of GPs

The flow of information from the department to the GP was typically outlined, either when the GP acknowledged the discharge summaries or when the oncologist asked. Furthermore, GPs pointed out their previous experience in, for example, pain treatment and expressed confidence in handling it. Their confidence in handling the task, therefore, became evident to the patient. Furthermore, previous contact with their GP was sometimes elaborated by the patients:

*'I would like to contribute as far as I can, because I have also been working at the Department of Oncology for several years, so I have some experience.'* (GP02)

#### ​Shared decision-making

Sharing needs and concerns with both doctors facilitated patient decisions about cancer and complementary treatment. The GP supported the oncologist in explaining the decision for the patient and how a specific treatment influenced the patient. Likewise, both GPs and oncologists supported a decision about follow-up in general practice regarding psychosocial issues related to having cancer. Hence, decisions were made in a tripartite coalition between the patient and the two doctors:

*'But if I may interrupt, what I was thinking was that you have pain now, and you have pain. You do not have many painkillers on your list of medicines. After all, the idea was, or the hope is, that the radiation therapy can help on your pain.'* (GP10)

*​'What would you* [GP] *suggest, what would you* [GP] *say.'* (P07)

#### ​Clarification of roles and responsibilities

As patient problems and needs were identified, patients, oncologists, and GPs took on different tasks, by which different roles relevant to the cancer trajectory emerged. Patients confirmed their responsibility to respond to signs and symptoms of impairment and cancer recurrence, to have blood samples taken, and book follow-up by the GP. GPs refrained from making an appointment right away but asked the patient to book later. Work-related issues and professional support for psychosocial issues were always a task assigned to the GP. The role of oncologists revolved around the cancer treatment; for example, when to change, continue, end, or re-evaluate the oncologic treatment, and taking care of side effects from treatment:

*​'I'll come to a home visit next time. I just made the booking into my calendar on the … approx ... Should I send you an email with the date?'* (GP12)

*​'I think you should try to talk to our secretary because first of all, it depends on your schedule.'* (GP10)

*​'It's nice to know where to place the ball.'* (P01)

### Category 2: Consultation structure

Three themes were grouped into this category: ‘setting an agenda’, ‘patient-centredness’, and ‘building a relationship’.

#### ​Setting an agenda

The oncologists invited patients and GPs for the consultations, and in line with the consultation guide, they naturally took on a leadership role. As people were sitting apart, the welcoming procedure involved a formal presentation of the doctors. In cases where the patient was sitting with the oncologist, the GP often initiated the dialogue by greeting the patient by their first name in an informal way. Afterwards, the oncologist introduced themselves and the patient. After the introduction, the GP typically introduced themselves by name and some comments about the relationship with the patient and previous contacts during the trajectory. Attending relatives were not always introduced, and only rarely was the assisting nurse who always accompanied the oncologist. Neither relatives nor nurses were visible on the video screens. The oncologist elucidated the purpose of the consultation. After a summary of the cancer treatment, the patient and the GP were sometimes asked if they had topics to be discussed.

Towards the end of almost all consultations, the oncologist asked if the patient or GP had further comments, and if so, the agenda was repeatedly agreed on. GP questions often centred on issues to be aware of, whereas patient questions most often regarded future treatment and follow-up:

*​'Well, but the idea of this is that we have to see things from these three perspectives. Especially the main character, but then also a little bit, how it looks from the general practitioner, and how it looks from the hospital.'* (Onco11)

*​'Then we have just a few principles for who takes care of what, and we have made some concrete agreements regarding Monday.'* (Onco12)

*​'What about the blood test for liver function, because I think they are rising? Is that something you* [oncologist] *have some comments on?'* (P11)

#### ​Patient-centredness

The doctors allowed the patients to provide input and participate actively, without the use of technical terms. Both GPs and oncologists participated in engaging the patients in the dialogue by encouraging them to express their needs and concerns, and by giving time to reflect and explain. The consultations were started by addressing the patient as ‘the key person’ and by explaining the purpose of the consultation. Both doctors participated in framing the various information and in sharing decisions with the patient, all without the use of technical terms. Emotions were responded to by GPs and oncologists by acknowledging and expressing support:

*​'Can you try to put into words how you feel at the moment?'* (Onco03)

*​'How does it look for you* [patient]*, is there anything special you have thought about today, now that you* [patient] *have both your doctors here?'* (Onco11)

*​'I do not know, here before today, are there some special things you have thought of, we should bring forward, now that both a doctor from the hospital and your family doctor is here?'* (Onco07)

#### ​Building a relationship

Relationships were built or reconsidered between patient and GP, patient and oncologist, and between the two professionals. Relationships between GPs and oncologists were primarily achieved through mutual support of decision-making, sharing thoughts regarding treatment or potential side effects, and by acknowledging each other’s expertise. The oncologist initiated the patient–doctor relationship for a consultation by explaining the mutual agenda. Furthermore, it was observed that both GPs and oncologists participated in building a doctor–patient relationship by developing rapport and providing summaries repeatedly. In most consultations, the oncologist summarised the consultation with both internal and concluding summaries, including decisions on future care.

### Category 3: Health concerns

This category covers the medical as well as psychosocial-driven content of the consultations. In line with the consultation guide, eight themes emerged and finally constituted this category: ‘physical wellbeing’, ‘side and late effects’, ‘comorbidity’, ‘medicine’, 'psychological wellbeing’, ‘social problems’, ‘relatives’, and ‘return to work’. Not all these themes emerged in every consultation, but were all addressed in several situations, implying their relevance. The doctors addressed themes clustered around treatment while psychosocial problems were brought up equally by patients, oncologists, and GPs. Psychological wellbeing and comorbidity were the themes most often leading to follow-up in general practice. The challenges faced by relatives were often not articulated, and when asked about, it was most often by a GP knowing them from previous visits:

*'* *You are always welcome if there is something that troubles you* *.* *'* (GP07)

*'* *A patient who has different health challenges with diabetes and heart surgery. So, it has been intense over the past few years with different things that have to be overcome* *.'* (GP08)

## Discussion

### Summary

This study is the first investigating consultations bringing a patient with cancer in ongoing oncology treatment together with the oncology specialist and GP in a cross-sectoral, multidisciplinary video consultation. Based on recordings and audio transcripts of 12 video-based consultations, it has been demonstrated that the content and structure of the consultations succeeded in being noticeably patient-centred. The consultations led to information exchange believed to contribute to professional learning. Articulation of the knowledge level of GPs may have convinced the patients that their GPs could take on the responsibility of supportive treatment such as pain management. Thereby, the tasks were clarified and shared. Psychosocial wellbeing and comorbidity were the themes most often leading to follow-up visits in general practice.

### Strengths and limitations

Drawing the cases consecutively from the intervention group of a RCT,^23^ the study succeeded in obtaining a sample that was representative of patients, oncologists, and GPs. Furthermore, patients were able to be present on screen with the oncologist and GP consultation. Saturation of themes was reached during the analytical phase. However, potential limitations need to be taken into consideration when interpreting the study results. The observations were based on a research setting and the study’s point of origin, Vejle Hospital, which is known for being innovative and open-minded regarding cross-sectoral cooperation,^29^ shared decision-making,^30^ and patient-centred communication.^31^

The research group represented a wide variety of medical and research experience, which was fruitful during the analytical process.^28^ The group were engaged in designing the study, but when analysing the recordings, did not have information from the inter-group analysis of the RCT.

In addition to studying the health concerns from the consultations and in concordance with the consultation guideline, the authors wanted a strong focus on patient-centredness, and the interaction between the triad of two medical professionals and a patient. Therefore, the authors actively chose to analyse the recordings, and strengthened the analysis based on the framework method. The framework allowed for a combined method when deducting themes, thereby giving the opportunity to include the list of potential themes from the consultation guide (Box 1) and still bring an open mind to the analysis (Box 2). To avoid an observer effect, it was decided not to supplement the data with clinical observations.^32^ Interviewing the participating patients, oncologists, and GPs could have been another data source and this study is planned for the future.

### Comparison with existing literature

To the authors' knowledge, The Partnership Study is the first to report live video consultations including a patient in ongoing treatment and two medical professionals representing different sectors. Although not the same but a similar setup, MDTs have been acknowledged for years for treatment planning in oncology.^33^ Recent trials of MDTs have included GPs^13^ or patients,^34–36^ but none simultaneously. Research in MDT involving GPs highlights the opportunity for enhanced continuity of care and allowing the professionals to build relationships. When including patients in MDTs, no change of anxiety or depression levels has been observed.^36^ Sensitive communication style, presentation of all present specialists, and the limited use of technical terms have shown to be important.^34^ In line with these suggestions about essentials for communication and building professional relationships, the doctors succeeded having a patient-centred communication style. This suggests that their obvious patient-centred approach may have been fruitful for inclusion of patients’ influence in shared decision-making about future tasks and roles; that is, for succeeding in tripartite decision-making. A strong doctor–patient relationship is also essential for providing quality care.^37^ GPs often have a long doctor–patient relationship,^38^ which may help to accommodate the sensitive communication style.

Van Gurp *et al* ^39^ described a collaboration study aimed at establishing tripartite consultations in palliative care. The GP was invited to the patient’s home to attend a video-based consultation with a hospital-based palliative care specialist (nurse or doctor). However, Van Gulp *et al* ended with a study exploring ‘back-stage’ teleconsultations between GPs and palliative care specialists who individually had a prior one-to-one consultation with the patient. Only one out of 18 cases succeeded in having a tripartite consultation. In line with observations from this single case, a high degree of patient-centredness^19^ through willingness from both doctors was observed. It is known from previous studies that patient-centredness has been associated with better recovery from discomfort and concerns, better emotional health, and fewer diagnostic tests and referrals.^40–42^ It is, therefore, argued that showing the success of offering patient-centred consultations is of great importance to future cross-sectoral coordination of care. Furthermore, Van Gurp *et al* exemplified how professionals can lose the trust of patients and family caregivers by starting bilateral medical–technical discussions incomprehensible to patients.^39^ Similar results regarding medical–technical discussions were found in a study of patients attending MDT.^34^ In contrast, the health professionals in the current study only used medical–technical terms when discussing topics for mutual professional learning, which were relevant for the specific patient.

Cancer care is complex and logistically difficult to coordinate across disciplinary and sectoral boundaries.^4^ The clarification of tasks is a challenge, but an essential step in role clarification for health providers.^43^ Previous intervention and qualitative studies have shown that GPs value better oncology specialist and GP communication, and that role clarification is needed, together with greater mutual trust.^3,43–45^ In line with these demands, the results of the study indicate that doctors can address and share current as well as future tasks. Decisions were shared and agreed with the patient, taking into account the patient’s desire for information, meaning patient-centred care.^42^

Looking at the different health concerns of the consultation, the study found that work-related issues and professional support of psychosocial issues were always defined as GP tasks. Furthermore, comorbidities were the most common reason for agreement on a follow-up in general practice. These findings are in line with previous suggestions of the expanding role of primary care in cancer.^4^ Oncologist tasks revolved around oncological treatment, and acute and late side effects. The findings regarding role clarification are in line with a survey among patients with cancer, oncologists, and GPs by Cheung *et al*.^46^ They found that patients preferred their primary care providers to have a significant role in the treatment of comorbidities.

### Implications for research and practice

Use of video in health care and cross-sectoral communication are in high demand.^47^ This pioneering work showing that tripartite video consultations can be performed in a patient-centred manner and it is believed that facilitating the involvement of the GP improves cross-sectoral cooperation and continuity of care. The study may improve acceptability for future innovation and enrolment in similar trials. However, additional knowledge on user perspectives from participant surveys, interviews with participants and between-group analysis from the RCT are essential to sufficiently understand this new way of communication, and the partnership intervention in particular.^23^ Also, trials including other cancer hospitals and patient groups are important for understanding generalisability and usefulness.

Digital technologies are rapidly upgraded. Limitations in the video system could be a possible reason why relatives were only occasionally engaged. Wider-angled cameras and focus on the positioning may improve the involvement of relatives and underline the attendance of oncology nurses.

In contrast to referral letters, the consultations allowed the participants to receive feedback in real time and this suggests that in the future GPs can play an essential role in cancer care by contributing to information sharing and task clarification. The recognition of GP expertise from the oncologist is also a way to facilitate patient trust in the GP’s ability to handle cancer-related issues.

This study showed that when overcoming the organisational and logistical barriers, tripartite video consultations seem useful to the patient, educational to professionals, and a way of clarifying tasks between specialists, all in a patient-centred way.

## Trial registration number

ClinicalTrials.gov identifier: NCT02716168

## References

[1] Gilbert HV, Yan J, Hoffman SJ (2010). Framework for action on interprofessional education and collaborative practice.

[2] Grunfeld E, Earle CC (2010). The interface between primary and oncology specialty care: treatment through survivorship. J Natl Cancer Inst Monogr.

[3] Lawrence RA, McLoone JK, Wakefield CE, Cohn RJ (2016). Primary care physicians' perspectives of their role in cancer care: a systematic review. J Gen Intern Med.

[4] Rubin G, Berendsen A, Crawford SM (2015). The expanding role of primary care in cancer control. Lancet Oncol.

[5] Klabunde CN, Ambs A, Keating NL (2009). The role of primary care physicians in cancer care. J Gen Intern Med.

[6] McCabe MS, Partridge AH, Grunfeld E, Hudson MM (2013). Risk-based health care, the cancer survivor, the oncologist, and the primary care physician. Semin Oncol.

[7] Grunfeld E (2008). Primary care physicians and oncologists are players on the same team. J Clin Oncol.

[8] Harrison JD, Young JM, Price MA (2009). What are the unmet supportive care needs of people with cancer? A systematic review. Support Care Cancer.

[9] Holm LV, Hansen DG, Johansen C (2012). Participation in cancer rehabilitation and unmet needs: a population-based cohort study. Support Care Cancer.

[10] Holm LV, Hansen DG, Larsen PV (2013). Social inequality in cancer rehabilitation: a population-based cohort study. Acta Oncol.

[11] Grunfeld E (2005). Cancer survivorship: a challenge for primary care physicians. Br J Gen Pract.

[12] Vierhout WP, Knottnerus JA, van OOij A, Crebolder HFJM (1995). Effectiveness of joint consultation sessions of general practitioners and orthopaedic surgeons for locomotor-system disorders. Lancet.

[13] Pype P, Mertens F, Belche J (2017). Experiences of hospital-based multidisciplinary team meetings in oncology: an interview study among participating general practitioners. Eur J Gen Pract.

[14] Elbert NJ, van Os-Medendorp H, van Renselaar W (2014). Effectiveness and cost-effectiveness of ehealth interventions in somatic diseases: a systematic review of systematic reviews and meta-analyses. J Med Internet Res.

[15] Sabesan S, Allen DT, Caldwell P (2014). Practical aspects of telehealth: establishing telehealth in an institution. Intern Med J.

[16] Sabesan S, Allen D, Caldwell P (2014). Practical aspects of telehealth: doctor–patient relationship and communication. Intern Med J.

[17] Tates K, Antheunis ML, Kanters S (2017). The effect of screen-to-screen versus face-to-face consultation on doctor-patient communication: an experimental study with simulated patients. J Med Internet Res.

[18] Black AD, Car J, Pagliari C (2011). The impact of eHealth on the quality and safety of health care: a systematic overview. PLoS Med.

[19] Epstein RM, Duberstein PR, Fenton JJ (2017). Effect of a patient-centered communication intervention on oncologist-patient communication, quality of life, and health care utilization in advanced cancer: the voice randomized clinical trial. JAMA Oncol.

[20] O'Brien BC, Harris IB, Beckman TJ (2014). Standards for reporting qualitative research: a synthesis of recommendations. Acad Med.

[21] Haggerty JL, Reid RJ, Freeman GK (2003). Continuity of care: a multidisciplinary review. BMJ.

[22] King M, Jones L, Richardson A (2008). The relationship between patients' experiences of continuity of cancer care and health outcomes: a mixed methods study. Br J Cancer.

[23] Trabjerg TB, Jensen LH, Søndergaard J (2019). Improving continuity by bringing the cancer patient, general practitioner and oncologist together in a shared video-based consultation — protocol for a randomised controlled trial. BMC Fam Pract.

[24] Pedersen KM, Andersen JS, Søndergaard J (2012). General practice and primary health care in Denmark. J Am Board Fam Med.

[25] Silverman J, Kurtz S, Draper J (2005). Skills for communicating with patients.

[26] Vasileiou K, Barnett J, Thorpe S, Young T (2018). Characterising and justifying sample size sufficiency in interview-based studies: systematic analysis of qualitative health research over a 15-year period. BMC Med Res Methodol.

[27] Harris PA, Taylor R, Thielke R (2009). Research electronic data capture (REDCap) — a metadata-driven methodology and workflow process for providing translational research informatics support. J Biomed Inform.

[28] Gale NK, Heath G, Cameron E (2013). Using the framework method for the analysis of qualitative data in multi-disciplinary health research. BMC Med Res Methodol.

[29] Dahler-Eriksen K, Nielsen JD, Lassen JF, Olesen F (1998). Cross-sectional therapeutic programs — an example of a cooperative health care system. A review with comments. Ugeskr Laeger.

[30] Steffensen KD, Vinter M, Crüger D (2018). Lessons in integrating shared decision-making into cancer care. J Oncol Pract.

[31] Ammentorp J, Graugaard LT, Lau ME (2014). Mandatory communication training of all employees with patient contact. Patient Educ Couns.

[32] Asan O, Montague E (2014). Using video-based observation research methods in primary care health encounters to evaluate complex interactions. Inform Prim Care.

[33] Fleissig A, Jenkins V, Catt S, Fallowfield L (2006). Multidisciplinary teams in cancer care: are they effective in the UK?. Lancet Oncol.

[34] Myhre A, Agai M, Dundas I, Feragen KB (2019). "All eyes on me": a qualitative study of parent and patient experiences of multidisciplinary care in craniofacial conditions. Cleft Palate Craniofac J.

[35] Choy ET, Chiu A, Butow P (2007). A pilot study to evaluate the impact of involving breast cancer patients in the multidisciplinary discussion of their disease and treatment plan. Breast.

[36] Chaillou D, Mortuaire G, Deken-Delannoy V (2019). Presence in head and neck cancer multidisciplinary team meeting: the patient's experience and satisfaction. Eur Ann Otorhinolaryngol Head Neck Dis.

[37] Ha JF, Longnecker N (2010). Doctor-Patient communication: a review. Ochsner J.

[38] Merriel SWD, Salisbury C, Metcalfe C, Ridd M (2015). Depth of the patient-doctor relationship and content of general practice consultations: cross-sectional study. Br J Gen Pract.

[39] van Gurp J, van Selm M, van Leeuwen E (2016). Teleconsultation for integrated palliative care at home: a qualitative study. Palliat Med.

[40] Dwamena F, Holmes-Rovner M, Gaulden CM (2012). Interventions for providers to promote a patient-centred approach in clinical consultations. Cochrane Database Syst Rev.

[41] Stewart M, Brown JB, Donner A (2000). The impact of patient-centered care on outcomes. J Fam Pract.

[42] Stewart M (2001). Towards a global definition of patient centred care. BMJ.

[43] Mitchell GK, Burridge LH, Colquist SP, Love A (2012). General practitioners' perceptions of their role in cancer care and factors which influence this role. Health Soc Care Community.

[44] Guassora AD, Jarlbaek L, Thorsen T (2015). Preparing general practitioners to receive cancer patients following treatment in secondary care: a qualitative study. BMC Health Serv Res.

[45] Easley J, Miedema B, O'Brien MA (2017). The role of family physicians in cancer care: perspectives of primary and specialty care providers. Curr Oncol.

[46] Cheung WY, Neville BA, Cameron DB (2009). Comparisons of patient and physician expectations for cancer survivorship care. J Clin Oncol.

[47] Marshall M, Shah R, Stokes-Lampard H (2018). Online consulting in general practice: making the move from disruptive innovation to mainstream service. BMJ.

